# Investigating the geographic disparity in quality of care: the case of hospital readmission after acute myocardial infarction in Italy

**DOI:** 10.1007/s10198-020-01221-9

**Published:** 2020-09-07

**Authors:** Yuxi Wang, Simone Ghislandi, Aleksandra Torbica

**Affiliations:** Centre for Research on Health and Social Care Management (CERGAS), Department of Social and Political Science, Bocconi University, Via Guglielmö Röntgen 1, 20136 Milan, MI Italy

**Keywords:** Geographic variation, Readmission, Rehospitalisation, Italy, Quality of care, Length of stay, I11, I14, D63

## Abstract

Unwarranted variation in the quality of care challenges the sustainability of healthcare systems. Especially in decentralised healthcare systems, it is crucial to understand the drivers behind regional differences in hospital qualities such as unplanned readmissions. This paper examines the factors that influence the risk of unplanned hospital readmission and the geographic disparity of readmission rate in Italy. We use hospital discharge data from 2010 to 2015 for patients above 65 years old admitted with Acute Myocardial Infarction. Employing hierarchical models, we identified the patient and hospital-level determinants for unplanned readmission. In line with the literature, the risk of readmission increases with age and being male, while hospitals with higher patient volume and capacity tend to have lower unplanned readmission. In particular, we find that after patient risk-adjustments, there are differential effects of hospitalisation length-of-stay on the probability of readmission across the hospitals that are governed by different payment systems. For hospitals under a prospective payment system, the effect of length-of-stay in reducing the probability of readmission is weaker than hospitals under an ex-post global budget, but the overall readmission rates are the lowest. Moreover, there are substantial geographic variations in readmission rate across Local Health Authority and regions, and these variations of unplanned readmission are explained by differences in hospital length-of-stay and surgical procedures used. Our results demonstrate that differential hospital behaviours can be one of the potential mechanisms that drive geographic quality disparities.

## Introduction

In recent decades, welfare states are increasingly faced with significant challenges of keeping health expenditures under control while increasing the quality of the healthcare system. As a result, several countries have implemented healthcare reforms to increase decentralisation [1–5], to contain cost [6, 7], to favour patient choice and competition [8, 9], and to focus on measuring performance [10–12]. Institutions and health systems at various levels adopted different forms of governance strategies. However, the responsibility endowed at the sub-nation level and the quasi-market mechanism can potentially generate undesirable regional disparities in healthcare quality. As a result, an increasing body of literature has investigated the geographic variation in healthcare reimbursement and utilisation [13], hospital performance [14–16], and various other health outcome indicators [17, 18].

The challenge of quality variation is especially salient in Italy. The country is not only characterised by a persistent regional economic divide between the North and the South, several regions that accumulated a large amount of fiscal deficit during the financial crisis had to adopt strict cost-containing measures to control for their financial problems [19]. The tightened budget imperatives in a decentralised system may, in turn, widen the differences in healthcare access, quality of care and overall health outcomes across regions. This fiscal burden can be further exacerbated by an ageing population, where a rise in healthcare expenditure is imminent. As the welfare state assumes a fundamental role in providing an equitable distribution of healthcare resources [20], considerable variation in the provision and the quality of care can be of grave concern. In this article, we aim to explore the determinants and the geographic variation of one important healthcare quality indicator—unplanned readmission—among the elderly population.

Unplanned readmission rate is considered an intricate quality indicator for hospitals and can be alarming for cost-conscious healthcare systems [21]. Unplanned readmission not only incurs unnecessary opportunity costs for the provider but also generates distress among patients, especially for frail elderly patients. Although there is extensive literature on the marginal effect of certain patient factors on unplanned readmission, very few studies have examined the hospital level factors and how they can explain the geographic disparities in quality of care. As systematic geographic differences in readmission rate can be alarming for the healthcare system, insights into the various determinants of unplanned hospital readmission and its variation are warranted.

The paper is structured as follows. We first justify our motivation by reviewing the related literature and the institutional background of the Italian National Health System. We then explain the method and the data used for the empirical analysis. Finally, the results highlight the geographic disparity of quality of care and potential drivers.

### Related literature

The conception of horizontal equity in health policy concerns the idealised scenario of equal treatment for equal need, or equality of access [22]. Inevitably, health and healthcare are unequally distributed across different segments of the populations, but not all health-related inequalities are *per se* inequitable [23]. Specific determinants such as demographic or hereditary factors may have differential marginal effects on health outcomes, but they do not contribute to inequity of health but instead represent the differential needs for healthcare. Since the provision of healthcare is generally considered to be a resource to meet these needs, the unequal distribution of access and quality of care across patients with the similar morbidity but seek care in different geographic areas militates against the notions of horizontal equity [23]. Factors that contribute to such inequality can be related to macro-level socioeconomic factors, provider behaviour, or lack of information on local needs that inadvertently harm a specific part of the population, causing an overall loss in welfare. As high and equitable quality of care is one of the core goals of most National Health Systems, a close examination of the unwarranted variation is needed when economic constraints become ever more salient.

In evaluating the quality of care and hospital performance, the literature has primarily focused on two main indicators—30 days mortality and readmission [24, 25]. While findings on mortality tend to be relatively consistent, the results on unplanned readmission, defined as rehospitalisation within 30 days from a previous discharge, and its determinants remain inconclusive. The most widely investigated factors related to unplanned readmission at the patient level include the hospitalisation length-of-stay (LOS) and individual characteristics such as disease profile, age, gender and education [7, 26]. The impact of LOS on the probability of readmission has mixed results, with some studies demonstrating a strong negative effect [27–30] and other findings have shown otherwise [31, 32]. Overall, LOS not only reflects patients’ clinical and demographic characteristics but also represents provider behaviour. Therefore, a positive relationship between risk-adjusted LOS and readmission implies that hospitals may have discharged patients prematurely that resulted in readmission, while a negative relationship means initial hospital stays reduced the risk of readmission [31]. The intricate relationship was further investigated by Carey [33], who demonstrated the trade-off effects between longer LOS and the expected cost of readmission for providers. The association between readmission and cost is also explored by various researchers [25, 34, 35]. However, we do not observe systematic patterns, and the differences of results may be attributed to contextual, disease area and timing differences. Research on the associations between hospital-level practices and readmission rate also highlighted the importance of organisational factors such as primary care pathways and surgical procedures used [36, 37].

While understanding the marginal effect of the individual and hospital determinants on readmission is crucial, examining how variations in these factors may explain the geographic inequality in readmission underlines whether such disparity reflects the heterogeneity in the needs of patients, or the provider and general healthcare delivery differences. We, therefore, connect the broader literature that investigates the variation of distinct dimensions of health and healthcare. Inter-regional disparities in resource allocation and efficiency of care are generally considered to be one of the main drivers of variation in the different dimensions of healthcare [38]. Some recent researches have looked at the variation in health and wellbeing indicators [39–41]; others have quantified the inter-regional variation in healthcare delivery and hospital performances [14–18]. The findings stress the importance of both patient and hospital factors variations in explaining the geographic difference in health-related outcomes.

This paper departs from these streams of literature and focuses on both the marginal effects of different determinants of unplanned readmission and the geographic disparity of this quality indicator. To our knowledge, this is the first investigation on how geographic variations of the patient and hospital factors are related the geographic disparities in quality of care in the Italian context. The findings have profound implications for the design of hospital incentive structures and the future resource allocation in the decentralised healthcare system.

### Institutional background

The Italian National Health System, which follows the Beveridge model since 1978, provides universal coverage to every citizen and is mainly funded through national and regional taxation [2, 19]. The Ministry of Health has an executive role over national health planning. At the same time, the organisation and provision of healthcare services are overseen by the 19 regions and 2 autonomous provinces and involves over 150 Local Health Authorities (LHAs or *Azienda Sanitarie Locali, ASLs*). Each Local Health Authority has an average catchment area of 437,000 people and is in charge of providing both primary and secondary care, as well as various independent public hospitals that administer tertiary care [42].

In the early 1990s, the Reform Law introduced decentralisation in the form of devolution in the Italian NHS, where the state gradually ceded its jurisdiction to its 20 regions. This process followed the international New Public Management [43] movement where organisational, political and fiscal devolution were encouraged to make regions more responsible for their health service activities and funding. Such decentralised feature is also present in many other European countries such as Denmark, Germany, Sweden and Spain [1]. In 2001, fiscal decentralisation to the regions was implemented (legislative decree 56/2000), and such constitutional reform in Italy endowed regions with the freedom to choose the type of healthcare model [42]. What was previously known as the Local Health Units (*Unità Sanitarie Locali*) were transformed into the current Local Health Authorities (LHAs), which directly run the public Hospital Units (HUs or *Ospedalia Gestione Diretta*) with their capitated budget and management [44]. Other hospital ownership types included Hospital Trust (*Aziende Ospedaliere*) that are granted the status of trusts with full managerial autonomy, Teaching Hospitals (*Clinici o Policlinici Universitari*), Research Hospitals (*Istituto di Ricovero e Cura a Carattere Scientifico, IRCCS*), Accredited Private Hospitals (*Case di Cura Accreditate*) and other private providers that compete with public hospitals in healthcare deliveries.

Regarding hospital care financing, regions have full autonomy to identify the services to be reimbursed through lump-sum, and to opt for their own diagnosis-related groups (DRGs) tariffs and funding schemes. Regional tariffs may be differentiated by the provider type to reflect the production costs and different responses to price incentives [44]. In general, public Hospital Units directly managed by LHAs are solely financed by global budgets that are based on the consumption of production factors such as personnel, and goods and services. Their budgets are kept separated from the overall budget of LHA’s, but their expenses are fully covered within the LHA’s financial resources retrospectively [44]. Therefore, Hospital Units do not necessarily have the financial incentives to attract patients and have less pressure to discharge patients early to reduce costs. In contrast, all other types of hospitals are financed primarily by the DRG-based Prospective Payment System (PPS). Under PPS, hospitals are reimbursed a fixed tariff per hospitalisation stay until a certain threshold of LOS, and the unit tariff decreases beyond this threshold to incentivise greater efficiency. For inpatient care provided by the independent public hospitals such as Hospital Trust and Teaching Hospitals, the reimbursements are based on two main components: activity-based payments according to the DRG-classification of discharges and a lump-sum based on average production costs for specific services such as emergencies and management of chronic illness. While for private accredited hospitals, funding is almost entirely dependent on PPS related allocations. Moreover, all regions are free to discriminate tariffs across providers to approximate the price to the actual costs and local specificities.

Following the devolution process in early 2000, some regions capable of executing the reforms experienced improvements in their systems, while others with weaker managerial capacity gradually worsened their financial sustainability [45, 46]. Tighter cost-containment measures further exacerbated the imbalance in light of the recent economic crisis [19]. Between 2001 and 2010, ten regions (Abruzzo, Molise, Apulia, Campania, Calabria, Sicily, Lazio, Piedmont, Sardinia and Liguria) consequently accumulated significant deficits and were expected to reduce the problem of cost over-run [47]. In practice, providers in these regions may reduce the number of beds, the number of staffs or patients’ length of hospitalisation.

Consequently, the governance of the NHS is divided into two regional clusters: those with stronger financial capacities retained some health policy autonomy, while the weaker regions were subject to strict central control [42]. For instance, the Lombardy region provides outcome benchmarking and splits purchasers and providers to encourage patient choice and competition [11]. At the same time, many southern regions such as Apulia, Campania, Calabria and Sicily employ a ‘command and control’ model with an active role of performance management [11]. There is persistent variability of the regional governance models in terms of the managerial structure of hospital care and the extent to which accredited private hospitals are involved in the provision of services [44]. Although there is a significant reduction in the regional deficit and increased stability of the NHS budget to date [42], the consequence on the quality of care remains unclear. Given the high variation in the financing and provision of healthcare services as well as the recent pressure to contain healthcare expenditures, Italy presents an intriguing case study to explore the factors related to geographic disparities in quality of care.

### Motivation and objectives

Our interest in the unplanned readmission indicator has two broad rationales: early hospital readmission represents an economic and social burden for cost-conscious healthcare system; it is subject to opportunistic behaviour [48] where providers discharge patients prematurely to reduce index hospitalisation cost or readmit a patient after a short time to get more reimbursement. The intricate nature of early readmission, therefore, indicate not only the quality of care but also the incentive structures of healthcare providers. Although not all readmissions are avoidable, low readmission rates are commonly regarded as the outcome indicator for good inpatient care [49]. Another widely used hospital performance indicator is the 30 days mortality after discharge. However, we do not have linked registry data and thus do not observe if the patient dies after discharge.

Our objectives are twofold: (i) to explore the marginal effects of factors related to the patient risk of readmission, (ii) to examine how hospital behaviour relates to the geographic variation of unplanned readmission rate. We pay specific attention to the hospital incentive structure, the discharge decision and the differential use of medical procedures and their role in explaining the geographic differences in readmission rates. The results provide important insights into the incidence and determinants of hospital readmission in Italy and the state of healthcare quality disparity for the observed years.

## Method

### Data

#### Study population

We analyse the hospital discharge data (Schede di Dimissione Ospedaliera, SDO) from the National Ministry of Health for the years 2010–2015. The data is routinely collected by all hospitals in all the regions and include not only administrative information such as diagnosis, treatment, discharge units, admission and discharge dates but also socio-demographic characteristics of the patients. Information about the hospitals in this dataset includes the type of ownership and the Local Health Authorities (LHAs) the institute belongs.

We focus on the elderly population because researches have found that patients over 65 years old are frail and at increased risk for readmission [50, 51], and that the Italian society is characterised by an ageing population suffering from a number of chronic conditions [37]. Moreover, patients were excluded from the analysis if any of the following criteria were met:

1. Patients who died during the hospital stay because they do not experience rehospitalisation.
2. Patients who are not admitted to acute care units, such as to rehabilitation or long-term-care unit, and therefore have very long length-of-stay.
3. Patients who are admitted through scheduled hospitalisation or transferred from other institutions, and thus readmission is planned.

In cases where patients incurred more than one admission during the first 30 days after discharge, we consider only the first readmission episode.

We select the patients diagnosed with a heart attack - Acute Myocardial Infarction (AMI) given the high volume of emergency admissions and that AMI patient unplanned readmission is commonly used as a healthcare quality indicator. We extract all patients whose main pathology is coded 410.0–410.9 under the 9th International Classification of Disease (ICD-9). Since these patients are often sent to the hospitals nearby, the potential selection bias is ameliorated when investigating the effects of geographic factors [52]. The treatments of AMI patients include Coronary artery bypass graft (CABG) or coronary bypass surgery, cardiac catheters, percutaneous transluminal coronary angioplasty (PTCA) and stent. CABG involves taking a vein or an artery from the patient’s body and using it to reroute blood from coronary arteries. A catheter is a thin, flexible tube that is inserted in a vein. PTCA is a minimally invasive procedure that uses an inflated balloon in a vessel to expand the blood vessel to improve blood flow, while the stent is a spring-shaped prosthesis used to complement PTCA. We extract the procedural codes from our dataset and control for the different interventions performed.

We also include organizational factors of the hospitals, such as the type of institution, capacity, and generic quality in the analysis. From the SDO data, we retain the hospital ownership type variable, which includes public Hospital Units (HUs or *Ospedalia a Gestione Diretta*), Hospital Trust (*Aziende Ospedaliere*), Teaching Hospitals (*Clinici o Policlinici Universitari*), Research Hospitals (*Istituto di Ricovero e Cura a Carattere Scientifico, IRCCS*), Accredited Private Hospitals (*Case di Cura Accreditate*) and other private providers. We calculated the volume of AMI patients per year by the provider from the SDO data. The information on the total bed counts of hospitals across the years is obtained from the Italian Ministry of Health (*Ministero della Salute*) website. The rationale for including the capacity information is to proxy the potential size constraints that hospitals face, which can be related to the readmission outcome. We also use the cut-off points of low, medium and high Acute Myocardial Infarction (AMI) mortality rate defined by the National Outcome Programs (Programma Nazionale Esiti) website for the broad quality categorization for the hospitals.

#### Outcome measure

The study’s primary outcome measure is the risk of readmission within 30 days after discharge for elderly patients diagnosed with Acute Myocardial Infarction (AMI) during the index hospitalisation. The primary outcome measure included readmission with all causes such as infections or complications, not just those that appear related to the initial admission. This measure is in line with the established literature and the readmission measure from the US Centers for Medicare and Medicaid Services and the QualityNet reporting guideline. In addition, because comorbid elderly patients may be more likely to be readmitted to the hospitals due to different pathologies, we also consider a more restricted definition of readmission that includes only readmissions with the same Major Diagnostic Category (MDC). For the analysis on the patient level, we consider these two types of readmission as binary variables to identify the effects of other explanatory variables. In estimating the geographic variations, we treat the readmission rates of each hospital as the outcome variable. The specifications are described in the following section.

Although unplanned readmission is a widely used quality indicator [21, 53] and represents substantial social and economic burdens, we are aware of some of the limitations of this indicator. First, adjustment for patient case-mix and contextual factors need to be carried out correctly to infer risk. We used the Ontario AMI prediction rules, a disease-specific instrument, to adjust for the risk scores of the patients. Second, studies show that not all readmissions within 30 days are avoidable [54], which can potentially make the indicator inaccurate. In recognising the potential weakness of the readmission indicator, we believe that the intricate nature of hospital readmission nonetheless offers important insights on the behaviours of the providers.

### Econometric specifications

Geographic disparities in unplanned readmission are linked to factors from various levels. First, differences in the local profile of the patients (case-mix) can be relevant if there is geographic sorting of, for instance, demographic characteristics. Second, at the hospital level, we consider organizational factors such as the type of ownership and capacity. Third, the influence of the Local Health Authority (LHAs)—specific random effects can contribute to the homogeneity within each of the healthcare market structures and the potential inter-LHA disparity in readmission rate. Finally, regional governments have considerable autonomy over their healthcare provision and fiscal policies, so the random effects at the regional level should also give rise to geographic variations. We thus need to account for the hierarchical geographic structure.

Given the multiple sources of variability, we identified two most relevant models in the literature: hierarchical generalized linear model (HGLM) and Cox proportional model with mixed effects. In fact, in a recent systematic review on the influence socioeconomic factors on hospital readmission for heart failure and AMI, most of the studies used either Cox proportional hazard regression or multivariate logistic regression [55]. The HGLM such as multilevel logistic model is commonly used to predict risks or odds ratios for readmission, while the Cox regression model with mixed effects, or sometimes called the frailty model [56], is a flexible model that accounts for the time until the failure event. As the two models are similar by construct and both explicitly model separate random effects at each level [57], we will employ both to understand how patient- and hospital-level variables affect the probability of early readmission.

To quantify the magnitude of the general contextual effect and variances at higher geographical levels, we aggregate the data to hospital level and estimate a linear multilevel mixed-effect model. We also estimate the intra-class correlations at different levels and the explained variance. We now describe each model in more detail.

#### Unplanned readmission and its determinants

We estimate both the multilevel logistics model for the probability of readmission, and multilevel proportional hazard model for time-to-readmission. As the healthcare path of the patients may depend on the structures of the providers and LHAs, we allow observation within the same hospital and LHA to be correlated to each other. As such, we are accounting for the within-cluster homogeneity.

For the multilevel logistics model, we estimate the following:

1 $$\begin{aligned}&\text {Logit}\;(\text {Pr}(Y_{ijk}=1))=\beta _0+\beta _{\mathrm{los}}\text {LOS}_{ijk}+\beta _{i}\text {LOS}_{ijk}\cdot \text {Type}_{jk}\nonumber \\&\quad +\,\beta _x{\bf{X}}_{\textit{ijk}}+\beta _z{\bf{Z}} _{\textit{jk}}+\mu _{t}+\mu _{R}+\beta _{R}\text {Inc}_{l} +e_{0k}+\eta _{0jk}+v_{0ijk} \end{aligned}$$

where $Y_{ijk}$ is the binary variable of patient *i* in hospital *j* in LHA *k*, and $Y_{ijk}=1$ if the patient is being readmitted to the hospital within 30 days of discharge. Here, each LHA cluster $k=1\ldots n$ consists of hospital clusters $j=1\ldots n_{i},$ and each hospital has $i=1\ldots n_{ij}$ patient observations. ${\bf{X}}_{\textit{ijk}}$ is a row vector containing the patient-level variables including demographics, comorbidities and LOS, and ${\bf{Z}}_{\textit{jk}}$ represents a vector of hospital-level factors such as capacity and patient volume. We allow for a non-linear relationship between age and our outcome variable by including a quadratic term. As discussed in the institutional background section, providers can have different discharge incentive structures due to their payment system. We thus interact the variable $\text {LOS}_{ijk}$ with the categorical variable of hospital types, $\text {Type}_{jk}$, to allow for the potential heterogeneous effects. We also include a set of year and regional fixed-effects ($\mu _t$ and $\mu _{R}$), as well as a regional average income variable $\text {Inc}_{l}$ to account for the economic disparity across regions. $\beta _x$, $\beta _z$ and $\beta _i$ are the fixed effects for the explanatory variables. Finally, $e_{0k}\;\sim \;N(0,\;\theta _e^{2})$, $\eta _{0jk}\;\sim \;N(0,\;\theta _\eta ^{2})$ and $\;v_{0ijk}\;\sim \;N(0,\;\theta _v^{2})$ are the random error terms at the LHA, hospital and patient levels, reflecting the cluster-specific random effects. We estimate the marginal effects of the explanatory variables through maximum likelihood. However, we do not report the intra-class coefficient (ICC) in quantifying the contribution of area-level variance to total variance because the computation and interpretation of ICC are often questionable in the context of logistic regression [58, 59].

Similarly, for the multilevel survival analysis, the underlying equation is:

2 $$\begin{aligned} h\;(t_{ijk})\;= & {} \;h_0(t)\;\cdot \;exp\;(\beta _{\mathrm{los}}\text {LOS}_{ijk}+\beta _{i}\text {LOS}_{ijk} \cdot \text {Type}_{jk}+\beta _x\bf{X}_\textit{ijk}\nonumber \\&+\beta _z{\bf{Z}}_\textit{jk}+\mu _{t}+\mu _{R}+\;\beta _{R}\text {Inc}_{l}+e_{0k}+\eta _{0jk}+v_{0ijk}) \end{aligned}$$

where $t_{ijk}$ is the observable failure (readmitted) time of the patient *i* nested in hospital *j* in LHA *k* and $h(t_{ijk})$ is the hazard function of the corresponding patient. $h_0(t)$ is the baseline hazard function. $\beta _x$, $\beta _z$ and $\beta _i$ are the conditional hazard ratios, while the remaining variables are the same from Eq. (1). This more flexible model is semiparametric and thus does not have a functional form assumption imposed on the baseline hazard. We estimate the model for the time from discharge to readmission and obtain the influence of the covariates at different levels.

#### Geographic variation of readmission rates

While it is important to identify the marginal effects of patient and hospital characteristics, we want to understand what drives the geographic variation in readmission. Since we are primarily interested in the unjustified variation generated from the providers, we aggregate the dataset to the hospital level while retaining patient variables as averages. The model consists of three geographic units—hospital, LHA and regions. As our outcome variable (hospital readmission rate) is no longer binary, we consider the multilevel mixed-effect linear model:

3 $$\begin{aligned} Y_{jkl}= & {} \beta _L\overline{\text {LOS}}_{\;jkl}\;+\beta _{i}\overline{\text {LOS}}_{jkl}\cdot \text {Type}_{jkl}+\beta _z{\bf{Z}}_{\mathit {jkl}}\;+\;\beta _x{\overline{\mathbf{X}}}_{\mathit {jkl}}\nonumber \\&+\mu _{t}+\mu _{R}+\;\beta _{R}\text {Inc}_{l}+\;\mu _{0l}\;+\;u_{0kl\;}+\;\varepsilon _{0jkl} \end{aligned}$$

where $Y_{jkl}$ represents the rate of readmission in hospital *j* of LHA *k* in region *l*, and $\overline{\text {LOS}}_{jkl}$ is the average LOS of the patients hospitalized in hospital *j*. ${\bf{Z}}_{\mathit{jkl}}$ represents the hospital ownership types, and the vector ${\overline{\bf{X}}}_{\mathit{jkl}}$ is the averaged patient-level information. $\mu _{0l}$ is the random intercept at the regional level, $u_{0kl}$ is the random intercept at LHA level, nested within region level, and $\varepsilon _{0jkl}$ captures the idiosyncratic hospital factors. We assume that $\mu _{0l}\;\sim \;N(0,\;\theta _\mu ^{2})$, $u_{jk}\;\sim \;N(0,\;\theta _u^{2})$ and $\varepsilon _{ojkl}\;\sim \;N(0,\;\theta _\varepsilon ^{2})$ and fit the model using restricted maximum likelihood for unbiased estimation of variances. We obtain the intra-class coefficients (ICCs) to assess the total residual variance attributable to both LHA and regional levels.

The total residual variance attributable to the LHA level is:

4 $$\begin{aligned} \text {ICC}_u=\frac{\theta _\mu +\theta _u}{\theta _\mu +\theta _u+\theta _\varepsilon }. \end{aligned}$$

And the total residual variance attributable to the regional level is:

5 $$\begin{aligned} \text {ICC}_\mu =\frac{\theta _\mu }{\theta _\mu +\theta _u+\theta _\varepsilon }. \end{aligned}$$

Larger values of ICC indicate that a considerable proportion of the residual variance in readmission rate is attributable to these levels. Visually, we compare the plots that rank the LHA and regional residuals for both the empty and the full models to assess the variation explained by the observed variables qualitatively.

We want to understand how much readmission variance is explained by differential hospital behaviours, here proxied by LOS and the different surgical procedures. This can be achieved by comparing the increase in explained variance after including the predictors. In the multilevel analysis, the presence of multiple variance components challenges the reporting of $R^{2}$ [60, 61] and we, therefore, calculate the proportional reduction in total variance after incorporating these predictors [62] using the following formula:

6 $$\begin{aligned} R^{2}\;(\text {S} \& \text {B})\;=\;1-\frac{(\theta _{\varepsilon ,\mathrm{{full}}}\;+\;\theta _{u,\mathrm{{full}}} +\;\theta _{\mu ,\mathrm{{full}}})}{(\theta _{\varepsilon ,\mathrm{{null}}}\;+\;\theta _{u,\mathrm{{null}}}+\; \theta _{\mu ,\mathrm{{null}}})\;\;}. \end{aligned}$$

We can argue that while, for instance, ownership-driven variation reflects organizational or structural disparities that are beyond the control of hospitals, inequalities that are driven by differential risk-adjusted LOS and the use of surgical procedures are arguably mitigable. If we observe a substantial increase in ${R^{2}}$ after the inclusion of LOS and innovative procedure, this implies the importance of discharge behaviour in driving the regional differences.

## Results

### Descriptive statistics

**Fig. 1:**
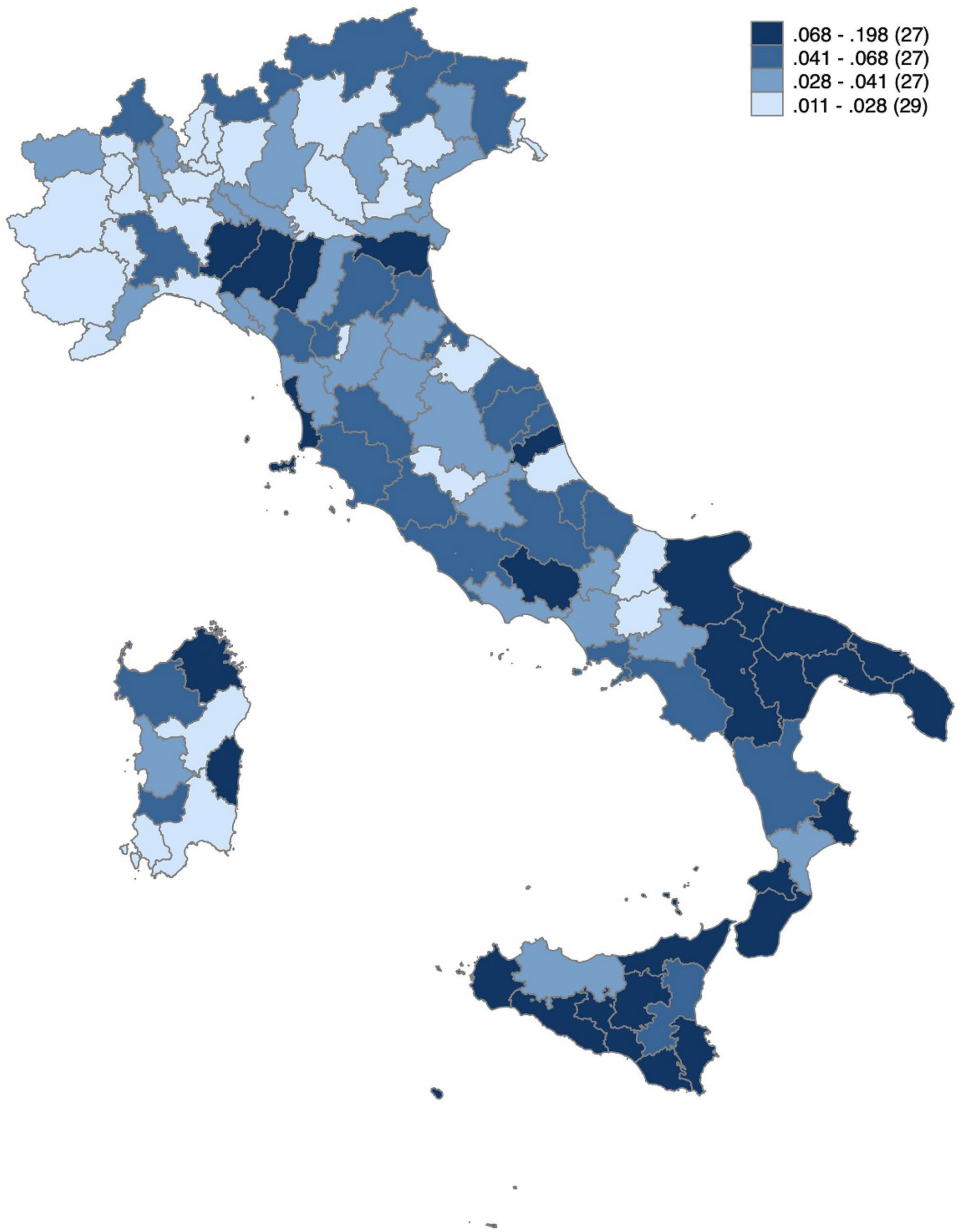
Average all-cause readmission rate by province, 2010–2015

Since we are examining the disparities across regions, we first compute the average rate of readmission in each region. Figure 1 shows the map of the provincial average all-cause readmission rates across all observable years. We can see that descriptively, the readmission rates differ across regions, with the northern regions having on average lower risks than the south. This difference reflects the general picture of the geographic disparity that characterize the economic development of the country.

In Table 1, we report all the patient- and hospital-level variables of the study population after our exclusion criteria. The patient-level data contains the age, gender, educational level, foreigner, LOS, the different intervention procedures, comorbidities and whether they were discharged to a rehab institution or integrated care home. At the same time, the hospital activity-related information includes volume, capacity, hospital type and AMI in-hospital mortality rate category (low, medium and high mortality) as a proxy for the hospital’s overall quality.

**Table 1:** Descriptive statistics

Characteristics of patients and hospitals			
Variables		Mean	SD
*Patient*			
Age		77.9	7.73
Male (%)		57.1	
Education level			
	Elementary school or lower (%)	24.83	
	Middle school diploma (%)	53.33	
	High school diploma (%)	15.02	
	University (%)	6.39	
	Laurea or above (%)	0.43	
Foreign (%)		1.1	
Length of stay (days)		8.91	7.78
PTCA and stent (%)		43.23	
Catheter(%)		1.04	
CABG(%)		5.5	
Ontario AMI comorbidities	Shock (%)	1.69	
	Diabetes with complications (%)	3.42	
	Congestive heart failure (%)	22.96	
	Cancer (%)	1.73	
	Cerebrovascular disease (%)	6.11	
	Pulmonary edema (%)	1.11	
	Acute renal failure (%)	2.52	
	Chronic renal failure (%)	10.7	
	Cardiac dysrhythmias (%)	17.7	
Discharged to institutions (%)		4.01	
Readmission within 30 days, all causes (%)		4.84	
Readmission within 30 days, same MDC (%)		0.67	
Observations	383,162		
*Hospital*			
AMI volume		77	101.16
Capacity		231	269.64
Types (#)			
	Hospital trust	109	
	Hospital unit	412	
	Teaching hospital	28	
	Research hospital	33	
	Private clinic	262	
	Others	39	
AMI mortality(#)			
	High	27	
	Medium	794	
	Low	62	
Observations	883		
*Region*			
Annual income (thousand)		29.64	4.34
Observations	21		

### Empirical results

#### Unplanned readmission and its determinants

**Fig. 2:**
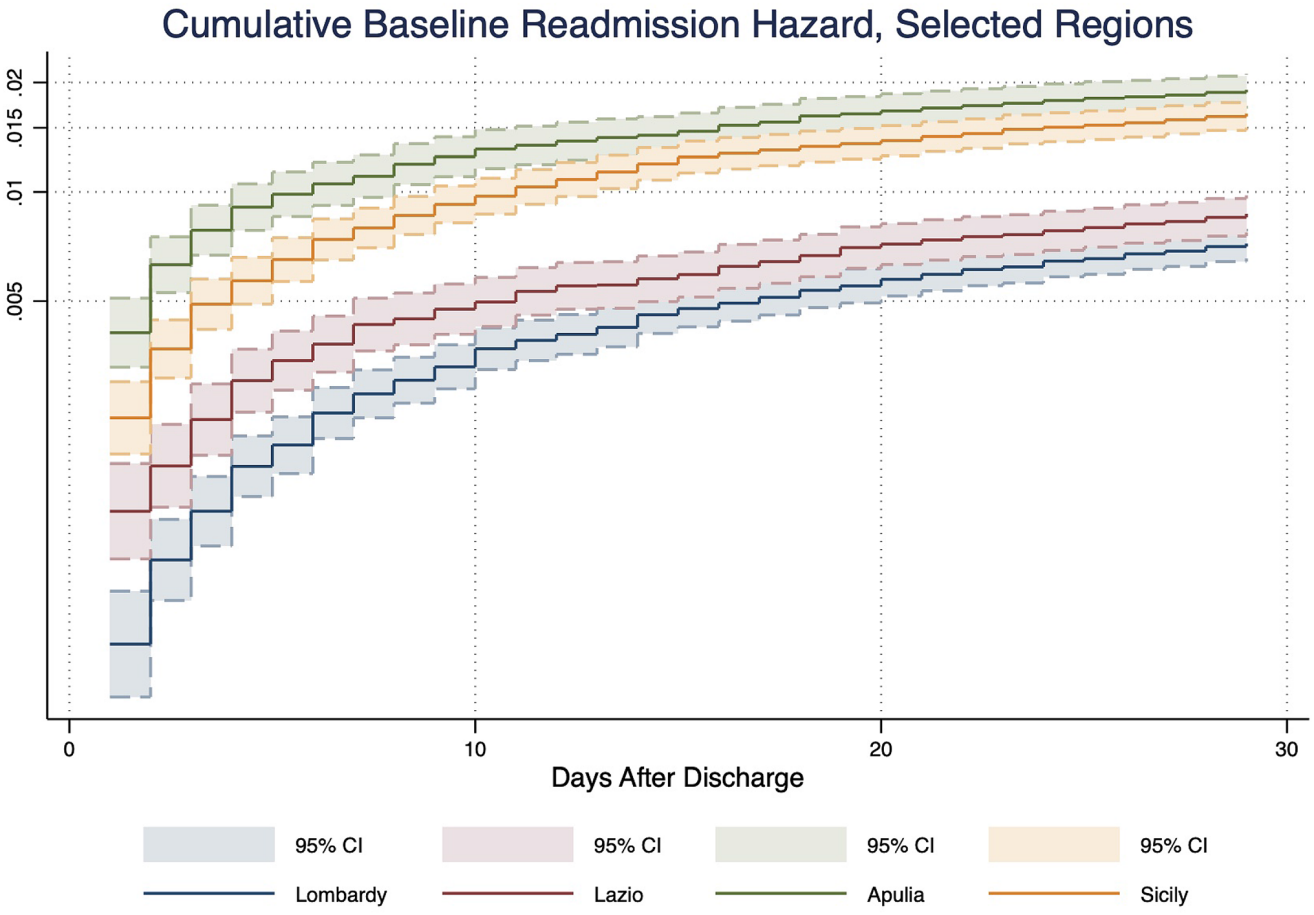
Cumulative baseline all-cause readmission hazard by selected regions

Before looking into the marginal effects of patient and hospital factors on readmission risks, we first present a descriptive graph of the readmission Nelson–Aalen cumulative hazard estimates as a function of days after discharge across the selected large regions, as seen in Fig. 2. We observe that the baseline readmission risk for patients who are admitted to hospitals in the Southern regions of Apulia and Sicily are significantly higher throughout the days after discharge than those admitted in Lombardy and Lazio.

Table 2 reports the coefficients of the patient and hospital-level variables from Eqs. (1) and (2). We analyse 30 days readmission for all causes as the primary dependent variable, as well as readmission with the same MDC as a secondary indicator. For all-cause readmission, we observe from the coefficients of the interaction term for LOS that, for both the multilevel Logit and hazard models, the probability to be readmitted decreases with LOS for patients admitted to all types of hospitals. The magnitude of this negative effect is higher for patients admitted to Hospital Units and Private Clinics than that of other hospitals. Moreover, the coefficients for hospital types show that independent public hospitals have significantly lower readmission probabilities than the Hospital Units and Private Clinics. This finding is particularly interesting as it partially relates to hospital incentive structures. For hospitals under a global budget as in the case of Hospital Units, there is little incentive to save costs, and thus the index hospitalisation LOS is more effective in preventing future unplanned readmission. Whereas for hospitals under a PPS with some budget allocations such as Hospital Trust, Teaching and Research Hospitals, the LOS is relatively less effective in reducing the probability of all-cause readmission given their incentive to improve efficiency. However, other than the effects of LOS, these independent public hospitals have significantly lower readmission risks than Hospital Units, indicating that other mechanisms other than payment systems are also driving the differences in readmission. Finally, for the profit-making private hospitals that operate solely under PPS, the effect of LOS in reducing the probability of readmission is the strongest, but they have the highest overall hospital readmission. For the more restricted outcome indicator, readmission with the same MDC, we observe a similar effect for LOS in terms of the directions of the coefficients. However, the coefficients are only significant for hospitalisations in Hospital Units and Private Clinics but not for the independent public hospitals.

For the demographic factors, the probability of readmission increases with age, but the effect diminishes with age. Males are more likely to be readmitted than females, and foreigners are less likely to be readmitted. Patients who underwent PTCA and Stent, CABG and Catheter all have less risk of all-cause readmission than patients with no operation performed. However, for readmission with the same MDC, the CABG procedure does not reduce the probability of readmission. The fact that patients were previously discharged to home hospitalisation, rehabilitation institution or other types of integrated home care does not affect the probability to be readmitted.

**Table 2:** Unplanned readmission and its determinants

Outcome indicator	All-cause readmission				Same MDC readmission			
Models	Logit		Hazard		Logit		Hazard	
Variables	Coefficient	SE	Coefficient	SE	Coefficient	SE	Coefficient	SE
*Patient-level*								
LOS ($\times$ hospital unit)	− 0.0765***	(0.00235)	− 0.0519***	(0.00240)	− 0.0717***	(0.00701)	− 0.0615***	(0.00845)
LOS $\times$ hospital trust	0.0319***	(0.00459)	0.0266***	(0.00454)	0.0158	(0.0126)	0.0202	(0.0148)
LOS $\times$ teaching hospital	0.0474***	(0.00530)	0.0338***	(0.00533)	0.00857	(0.0150)	0.00757	(0.0179)
LOS $\times$ research hospital	0.0475***	(0.0117)	0.0311***	(0.0118)	0.00639	(0.0387)	0.0199	(0.0401)
LOS $\times$ private clinic	− 0.0274***	(0.00988)	− 0.0199*	(0.0105)	− 0.0608**	(0.0262)	− 0.0825**	(0.0378)
LOS $\times$ others	0.0221**	(0.0107)	0.0228**	(0.0109)	0.0793***	(0.0237)	0.0913***	(0.0234)
Age	0.253***	(0.0210)	0.227***	(0.0229)	0.310***	(0.0594)	0.331***	(0.0758)
Age2	− 0.00173***	(0.000135)	− 0.00153***	(0.000147)	− 0.00189***	(0.000375)	(0.0757)	(0.000478)
Male	0.150***	(0.0169)	0.151***	(0.0185)	0.188***	(0.0490)	0.199***	(0.0622)
Foreign	− 0.239***	(0.0887)	− 0.170*	(0.0973)	− 0.808**	(0.357)	− 1.205**	(0.558)
PTCA stent	− 0.677***	(0.0226)	− 0.617***	(0.0247)	− 0.808**	(0.0608)	− 0.845***	(0.0786)
CABG	− 1.338***	(0.192)	− 1.466***	(0.228)	0.227	(0.270)	0.240	(0.340)
Catheter	− 0.267***	(0.0546)	− 0.270***	(0.0588)	− 0.405***	(0.144)	− 0.423**	(0.183)
Institutions	0.0401	(0.0613)	− 0.293*	(0.153)	− 0.242	(0.175)	− 0.210	(0.221)
*Hospital-level*								
Hospital type (reference hospital unit)								
Hospital trust	− 0.427***	(0.112)	− 0.367***	(0.0997)	− 0.113	(0.168)	0.0813	(0.219)
Teaching hospital	− 0.506***	(0.154)	− 0.381***	(0.137)	− 0.127	(0.196)	0.456	(0.287)
Research hospital	− 0.584***	(0.212)	− 0.415**	(0.198)	− 0.161	(0.240)	− 0.123	(0.505)
Private clinic	0.178*	(0.100)	0.00852	(0.102)	0.262	(0.172)	0.822***	(0.267)
Others	− 0.151	(0.160)	− 0.254*	(0.150)	0.254*	(0.150)	− 1.098***	(0.391)
AMI volume	− 0.0009***	(0.00025)	− 0.0008***	(0.00024)	− 0.00104**	(0.00044)	− 0.00127**	(0.00059)
Capacity	− 0.0006***	(0.00014)	− 0.0006***	(0.0001)	− 0.0009***	(0.0002)	− 0.001***	(0.0004)
AMI mortality								
Low	− 0.295***	(0.107)	− 0.0650	(0.100)	− 0.140	(0.170)	− 0.166	(0.238)
Medium	− 0.388***	(0.0929)	− 0.175**	(0.0863)	− 0.0154	(0.143)	− 0.0852	(0.201)
*Regional-level*								
Average income (thousand)	− 0.03**	(0.014)	− 0.03**	(0.015)	− 0.02**	(0.015)	− 0.01	(0.052)
Constant	− 10.98***	(0.944)	− 17.38***	(1.029)	− 17.63***	(1.038)	− 26.10***	(3.432)
ln_p			0.750***	(0.00872)			0.647***	(0.0265)
Variance LHA level	0.0702***	(0.0237)	0.0242	(0.0208)	0.0455***	(0.0171)	1.61e−09	(9.79e−05)
Variance hospital level	0.368***	(0.0321)	0.251***	(0.0429)	0.232***	(0.0226)	0.561***	(0.155)

Number of observations 383,162. Number of hospitals 883. Number of LHAs 154. Coefficients for comorbidities, education, regional and year fixed effects can be found in the Appendix Table 6

***Significant at 1%; **significant at 5%; *significant at 10%

At the hospital level, we have discussed that the independent public hospitals such as Hospital Trust and Teaching Hospitals have significantly lower risks of all-cause readmission than LHA-managed Hospital Units. However, the same effect is not observed for readmission with the same MDC. The volume of AMI patients reduces the probability of both types of readmission, indicating some degrees of learning effect. Furthermore, hospitals with higher capacity have lower probabilities of readmission. This effect is expected, as bed constraints may contribute to early patient discharges and in turn, result in unplanned readmission. Finally, patients admitted to hospitals with low and medium in-hospital AMI mortality (according to the National Outcome Programs) have lower likelihoods of all-cause readmission. The coefficients for comorbidities, education, years and regional fixed effects can be found in Appendix Tables 5 and 6. Overall, the probability to be readmitted has decreased with comorbid patients with Shock, cerebrovascular disease and Cardiac Dysrhythmias are less likely to be readmitted, while diabetic patients are more likely to be readmitted. This correlation for the above conditions can be explained by more considerable attention offered by the providers for patients with these severe cardiovascular comorbidities. However, the medical interpretation of the conditions is beyond the scope of this paper. In Appendix Tables 5 and 6, we also observed that all-cause readmission decreases over the years, but same -MDC readmission increases. Many of the Central and Southern regions have positive and significant coefficients, indicating higher general readmission risks.

#### Geographic variation of readmission rate

Hospital-level variation in the readmission outcome is estimated in terms of variance and intra-class correlation. We first present the coefficient estimates for the variables collapsed at the hospital level in Table 3. Although most coefficients have the same signs as in Table 2, some of them cease to be significant. Notably, for all-cause readmission rates, the coefficients for average LOS are significant for all types of hospitals except for Research hospitals, while for the same MDC readmission rate the coefficient is only significant for Private Clinics. Moreover, the percentage of patients who underwent PTCA and stent procedures have significantly negative coefficients for both types of readmission rate, while the percentage of CABG procedure only reduces all-cause readmission rate. These variables represent the underlying hospital behaviours and are robust to the aggregation (Table 3).

**Table 3:** Hospital readmission rate and its determinants

Models	All readmission		Same MDC readmission	
Variables	Coefficient	SE	Coefficient	SE
LOS ($\times$ hospital unit)	− 0.007***	(0.00123)	− 0.0007	(0.0005)
LOS $\times$ hospital trust	0.00493*	(0.00257)	0.000694	(0.00106)
LOS $\times$ teaching hospital	0.00916**	(0.00425)	0.000570	(0.00175)
LOS $\times$ research hospital	0.00380	(0.00291)	− 0.000442	(0.00120)
LOS $\times$ private clinic	− 0.00262*	(0.00148)	− 0.00163***	(0.000609)
LOS $\times$ others	0.000195	(0.00257)	0.000556	(0.00106)
PTCA stent (%)	− 0.168***	(0.0122)	− 0.0174***	(0.00499)
CABG (%)	− 0.148***	(0.0484)	− 0.0273	(0.0200)
Catheter (%)	− 0.00478	(0.0219)	− 0.000973	(0.00892)
Hospital type (reference hospital unit)				
Hospital trust	− 0.0440*	(0.0257)	− 0.00220	(0.0129)
Teaching hospital	− 0.0776*	(0.0442)	− 0.000545	(0.0182)
Research hospital	− 0.0326	(0.0289)	0.0117	(0.0120)
Private clinic	0.0504***	(0.0132)	0.0267***	(0.00540)
Others	0.0103	(0.0252)	− 0.00393	(0.0104)
AMI volume	− 6.33e−05*	(3.61e−05)	− 3.75e−06	(1.48e−05)
Capacity	− 5.70e−08	(1.29e−05)	− 2.00e−06	(5.35e−06)
AMI mortality				
Low	− 0.00909	(0.0145)	− 0.00183	(0.00595)
Medium	− 0.00456	(0.0123)	− 0.000356	(0.00506)
Average income (thousand)	− 0.0006	(0.002)	0.0002	(0.0004)
Constant	0.464***	(0.0771)	− 0.000738	(0.0281)

Number of hospitals 883. Number of LHAs 154. Number of regions 21. Coefficients for average patient characteristics and year fixed-effects can be found in Appendix Table 6

***Significant at 1%; **significant at 5%; *significant at 10%

**Fig. 3:**
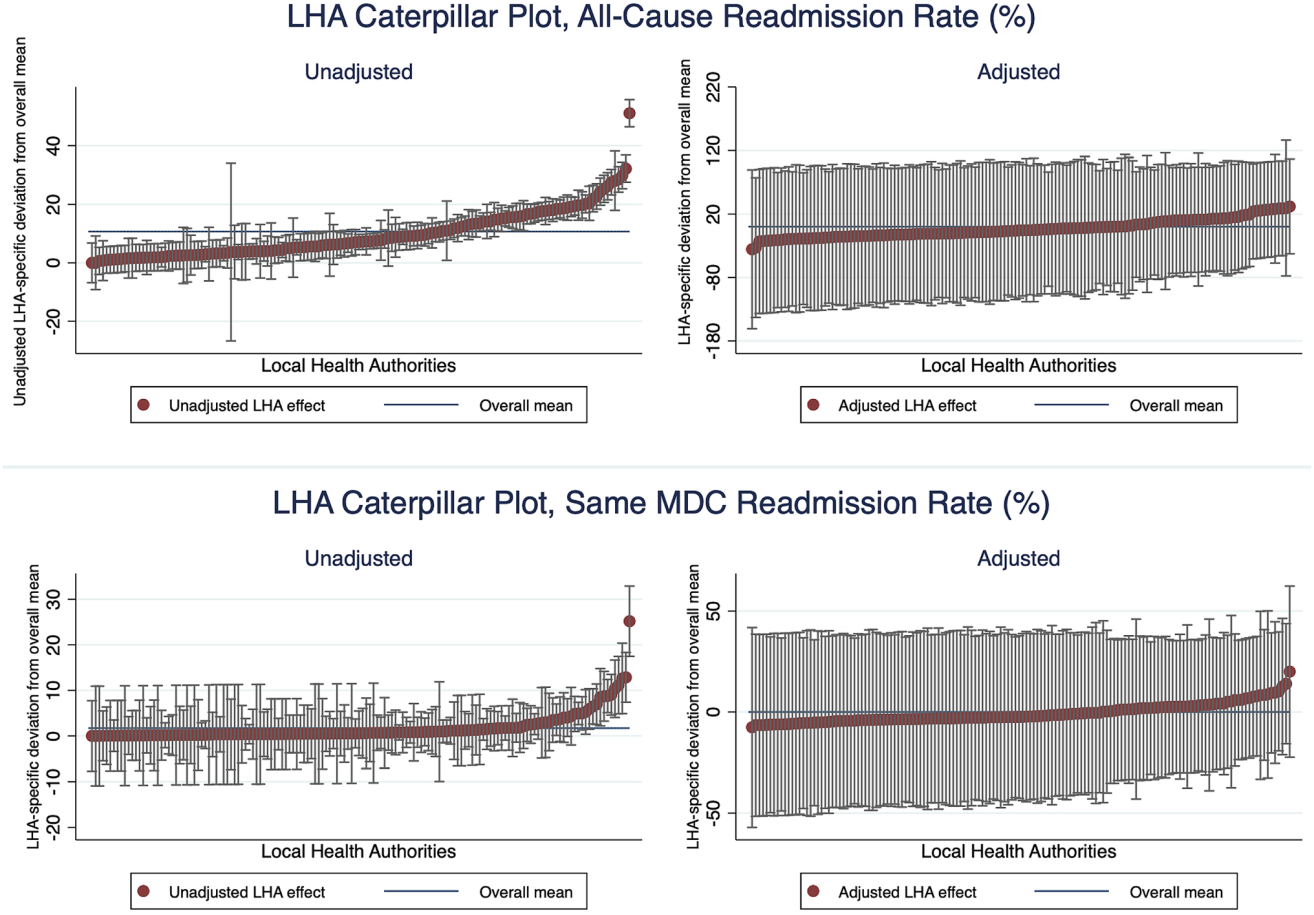
Local health authorities caterpillar plot, readmission rate

**Fig. 4:**
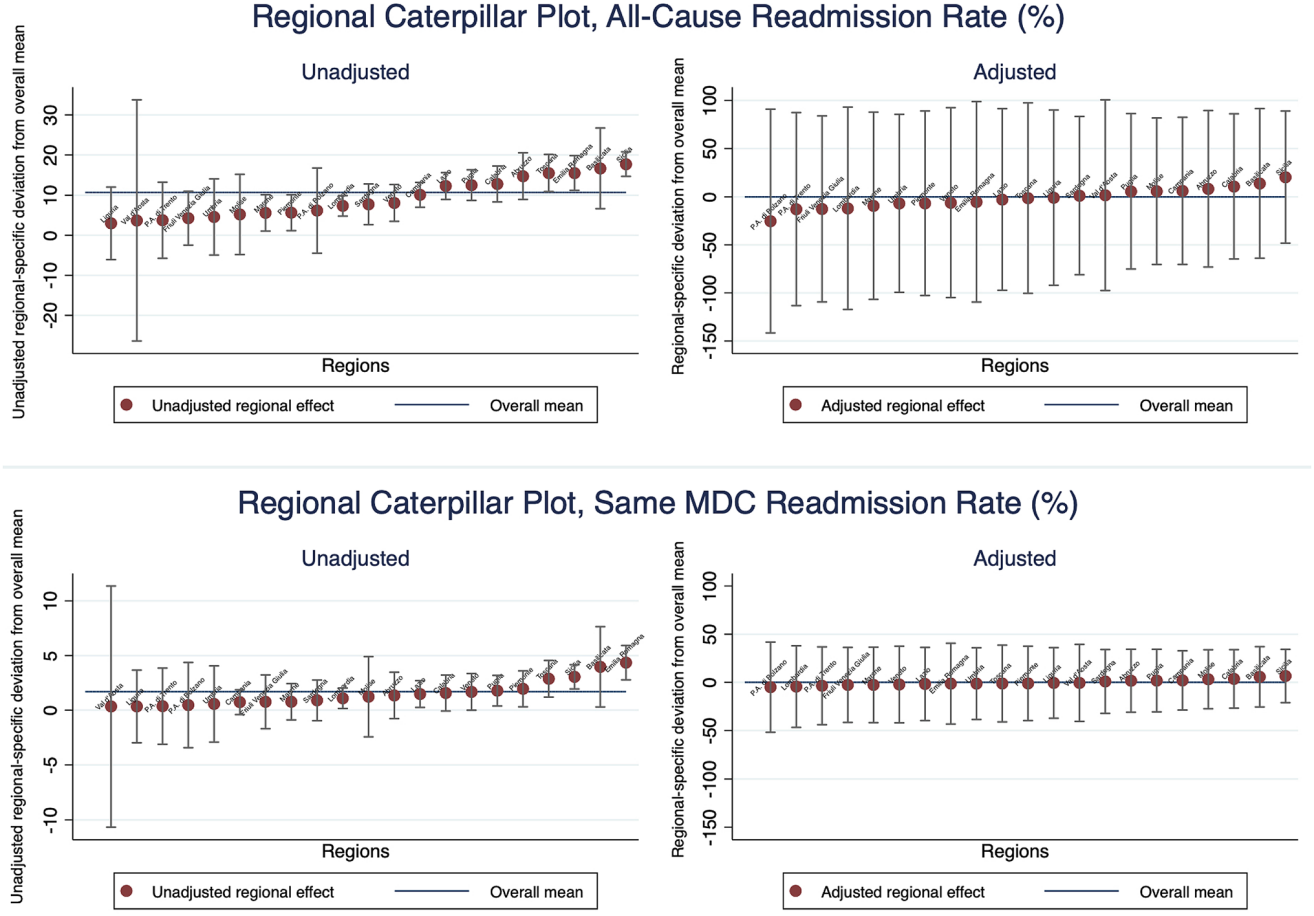
Regional caterpillar plot, readmission rate

We graphically represent the residuals from the empty and the full models at both the LHA and the regional levels. As seen in Figs. 3 and 4, we order the residuals by the unadjusted and adjusted LHA and regional averages of readmission rate and plot the 95% confidence intervals around each residual estimate. The adjusted residuals represent the unexplained variation after accounting for the differences across patient and hospital factors. We observe that, without accounting for the explanatory variables, some LHAs and regions exhibit significantly different levels of variation for both types of readmission rates. The variation is more pronounced for all-cause readmission, as we observe LHA and regions both significantly below or above the average readmission rates. In particular, we see in Fig. 4 Marche, Piedmont, Veneto and Lombardy have significantly lower-than-average regional all-cause readmission rate, while Emilia Romagna and Sicily has a significantly high readmission rate. After adjusting for patient and hospital characteristics, these variations diminished considerably.

Since we are interested in how hospital behaviours, here represented by LOS and different surgical procedures, explain the variation, we investigate the variance components in two separate models for both types of readmission rates. We further compute the intra-class correlation (ICC) and the explained variance as represented by Eqs. (4)–(6). The intra-class correlations (ICC) estimate the proportion of overall variation in outcomes explained by the variation between geographic units. As seen in Table 4, for the model excluding LOS and surgical procedures for all-cause readmission, we observe around 11.69% of the total variation is attributed to higher geographic levels, with about 7.89% at the LHA level and 3.8% at the regional level. After incorporating LOS and surgical procedures into the specification, the ICC decreased by 2.72% at the LHA-level and by around 0.4% at the regional level. At the same time the $R^{2}$ almost doubled from 0.1172 to 0.2175. This result indicates how LOS and the use of surgical procedures played an indispensable role in driving the geographic variation in all-cause unplanned readmission. Although the scale of results for same-MDC readmission rate is much smaller, we do observe that these hospital factors explained a considerable proportion of the overall variance at the LHA and the regional levels.

**Table 4:** Variance analysis

Models	Variance	ICC	R-square (S&B)
*All-cause readmission*			
Full model (exc. LOS and procedure)			0.1172
Hospital	0.02377		
LHA < region (154)	0.00106	0.0789	
Region (21)	0.00098	0.0380	
Full model			0.2175
Hospital	0.02169		
LHA < region (154)	0.00041	0.0517	
Region (21)	0.00078	0.0340	
*Same MDC readmission*			
Full model (exc. LOS and procedure)			0.04707
Hospital	0.00385		
LHA < region (154)	0.00003	0.02650	
Region (21)	0.00004	0.01210	
Full model			0.06567
Hospital	0.00379		
LHA < region (154)	0.00002	0.01479	
Region (21)	0.00004	0.00932	

## Discussion

In this article, we have investigated the determinants and the geographic variation of elderly hospital unplanned readmission during the period of a high level of decentralisation and cost-containing pressure. We have shown how differences in patient and hospital characteristics can contribute to the probability of readmission with hierarchical models. After accounting for sociodemographic and comorbidity variables, we found that the probability to be readmitted for all causes decreases with longer LOS for patients admitted to all types of hospitals. The magnitude of this negative effect is lower for independent public hospitals such as Hospital Trusts and Teaching Hospitals than for Hospital Units or Private Clinics. The use of PTCA and stent, CABG and catheter all decreases the probability of all-cause readmission, while the hospital AMI patient volume and capacity are both associated with lower all-cause readmission. Moreover, the effects of LOS, the different medical procedures and hospital types are relatively robust to aggregation to the hospital level. The results for readmission with the same MDC is comparable, while some coefficients lost significance. Our variance analysis further shows that there are strong contextual effects at the LHA and regional levels, while the variation in LOS and the use of different surgical procedures can explain a considerable proportion of the overall readmission variance. Our empirical results reveal the potential pathway through which readmission rates vary across geographic areas—differential provider behaviours.

Our findings on the patient-level determinants of readmission are broadly in line with the previous studies. Specifically, older and male patients are at increased risk of readmission, while longer LOS reduces the probability of readmission [26, 31, 34]. However, we uniquely contribute to the literature by incorporating more hospital-level factors and allowing LOS to have differential effects on readmission across hospital types. The findings reflect the role of hospital discharge incentives, which, to our knowledge, was never explored in previous research. In particular, since public Hospital Units in Italy are financed by global budgets that are reimbursed ex-post, we expect that they have less pressure to discharge patients early for cost-saving purposes. This is confirmed by the significant and negative coefficients of LOS for both of the readmission indicators. On the other hand, since the DRG-based PPS incentivises greater efficiency as reimbursement tariffs decrease beyond a specific hospital LOS, for independent public hospitals such Hospital Trusts, Teaching and Research Hospitals, there is more incentive to discharge the patients before the threshold date in order to avoid tariff abatement. Therefore, LOS may have been less effective in reducing the probability of all-cause readmission than the public Hospital Units. Nevertheless, these independent public hospitals have lower overall readmission than Hospital Units, which highlights the fact that payment incentive systems are not the only drivers of the different readmission rates across hospital types. We believe that future research can incorporate, both theoretically and empirically, the cost dimension of the provider behaviour in the Italian context, as explored in other countries by Kittelsen et al. [63] and Schreyögg and Stargardt [64].

Furthermore, our analysis of the geographic disparity of readmission rate is comparable to the research on the variation of hospital performance indicators such as emergency admission mortality [14, 65], LOS [16] and hospital resource utilisation [17] in other contexts. For instance, similar to our results, Gobillon and Milcent have found that differential use of surgical procedures contributed to the substantial regional disparity in AMI mortality in France [14]. In a multi-country analysis, Lorenzini and Marino have found that hospital size and types explain the cross-country variation in efficiency outcomes such as LOS and costs [16]. These studies highlighted the importance of understanding the disparity in healthcare delivery at different geographic levels. There are, however, two unique and important contributions from our findings. First, the geographic variation of unplanned readmission is primarily explained by not only differential procedures, but also hospital LOS. This result points to the potential geographic clustering of hospital discharge behaviour that can be important for policy-makers to improve equity of care. Second, the hierarchical geographic levels adopted in this paper are important units to consider given the highly decentralised healthcare system in Italy. Since LHAs are responsible for the health of the entire population in a given area, inter-regional differences in sources of funding, healthcare governance model may explain why, even after controlling for patient and hospital factors, we still observe around 10% of the total variance attributable to the LHA and regional level.

Some limitations of this paper need to be recognised. First of all, readmission as an indicator can be tricky to interpret, as there are variations of the percentage of readmission that is considered “preventable”, and reasons for early readmission also tend to differ substantially [66]. Secondly, since our dataset does not link to the registry data, we are not able to control or exclude the patients who died after discharge. Finally, we have not fully considered some of the contextual factors at the local health market, such as hospital competition and population density but instead treated them as cluster-specific random effects. This aspect will be essential to consider for future studies on the spatial distribution and patient travelling patterns for elective admissions.

## Conclusion

What we explored in this paper ultimately touches upon the trade-off between quality and efficiency and the potentially divergent trajectories of healthcare quality across regions. For hospitals under PPS, LOS may have been less effective in reducing all-cause readmission than that of hospitals are under a global budget system due to the lack of incentive to keep patients for longer than necessary. However, the overall readmission rates of these independent public hospitals remain significantly lower. Additionally, the negative effect of LOS is the strongest among the Private Clinics, which also have the highest overall readmission rate. These findings indicate that the differences in readmission risks across hospital types are not solely driven by payment incentives. For instance, even though we are analysing emergency admissions, patient selection may still be present in certain regions. The existence of private insurance and payments may also facilitate more extended hospital stay. In general, the geographic variations in unplanned readmission that are driven by differential discharge behaviour, surgical procedures or other unobserved factors had profound implications on the equity dimension of the healthcare system. For health policy-makers, it is admittedly a daunting task to achieve the right balance between endowing more autonomy to regions and maintaining a healthy level of central control over the quality of healthcare delivery. For instance, certain well-governed regions may have achieved both better quality of care and financial performance, while others struggle through the same period and remain at a stagnant stage where the financial constraint is limiting the progress to improve quality. Although after 2015, the cost containment measures have been eased in most regions, the variability across LHA and regions in terms of health governance models and the extent tariffs are used persists. We hope our findings can provide important insights into the potential driver of geographic disparity of quality of care.

## Appendix

See Tables 5, 6 and 7.

**Table 5:** Appendix, fixed effects, all readmission from Table 2

Models	Logit		Hazard	
Variables	Coefficient	SE	Coefficient	SE
*Education*				
Elementary or lower	Reference		Reference	
Middle school	− 0.0598**	(0.0251)	− 0.0463*	(0.0272)
High school	− 0.159***	(0.0326)	− 0.139***	(0.0359)
University	− 0.163***	(0.0434)	− 0.176***	(0.0484)
Laurea or above	0.0725	(0.118)	− 0.0270	(0.135)
*Comorbidities*				
Shock	− 0.286***	(0.0717)	− 0.358***	(0.0821)
Diabetes with complications	0.0565	(0.0435)	0.120***	(0.0461)
Congestive heart failure	− 0.0126	(0.0208)	0.00661	(0.0225)
Cancer	− 0.133**	(0.0655)	− 0.0852	(0.0697)
Cerebrovascular disease	− 0.217***	(0.0360)	− 0.199***	(0.0390)
Pulmonary edema	0.0771	(0.0780)	0.0796	(0.0839)
Acute renal failure	0.0502	(0.0573)	0.0558	(0.0612)
Chronic renal failure	− 0.000708	(0.0272)	0.0291	(0.0292)
Cardiac dysrhythmias	− 0.133***	(0.0223)	− 0.109***	(0.0241)
*Year fixed-effects*				
2010	Reference		Reference	
2011	0.0203	(0.0270)	0.0219	(0.0291)
2012	− 0.00896	(0.0307)	− 0.0366	(0.0334)
2013	− 0.0702**	(0.0322)	− 0.107***	(0.0352)
2014	− 0.0849**	(0.0332)	− 0.111***	(0.0361)
2015	− 0.180***	(0.0322)	− 0.225***	(0.0353)
*Regional fixed-effects*				
Piedmont (10)	Reference		Reference	
Aosta Valley (20)	0.616	(0.702)	0.747	(0.571)
Lombardy (30)	0.338*	(0.195)	0.245	(0.169)
P.A. Bolzano (41)	0.905**	(0.410)	0.709**	(0.354)
P.A. Trento (42)	− 0.208	(0.408)	− 0.257	(0.350)
Veneto (50)	0.379*	(0.200)	0.305*	(0.170)
Friuli Venezia Giulia (60)	0.101	(0.235)	0.255	(0.200)
Liguria (70)	0.255	(0.296)	0.263	(0.248)
Emilia Romagna (80)	1.189***	(0.198)	1.145***	(0.169)
Tuscany (90)	0.675***	(0.200)	0.577***	(0.168)
Umbria (100)	0.160	(0.250)	0.144	(0.213)
Marche (110)	0.481	(0.327)	0.495*	(0.269)
Lazio (120)	0.806***	(0.188)	0.614***	(0.159)
Abruzzo (130)	0.545**	(0.260)	0.345	(0.228)
Molise (140)	− 0.623	(0.485)	− 0.674	(0.441)
Campania (150)	0.258	(0.220)	0.0949	(0.197)
Apulia (160)	0.814***	(0.222)	0.635***	(0.194)
Basilicata (170)	1.333***	(0.355)	1.024***	(0.301)
Calabria (180)	0.619**	(0.251)	0.536**	(0.222)
Sicily (190)	0.735***	(0.232)	0.569***	(0.217)
Sardinia (200)	0.229	(0.237)	0.198	(0.206)

***Significant at 1%; **significant at 5%; *significant at 10%

**Table 6:** Appendix, fixed effects, same MDC readmission from Table 2

Models	Logit		Hazard	
Variables	Coefficient	SE	Coefficient	SE
*Education*				
Elementary or lower	Reference		Reference	
Middle school	0.0365	(0.0705)	0.00986	(0.0892)
High school	0.00916	(0.0915)	− 0.0196	(0.117)
University	− 0.135	(0.127)	− 0.215	(0.165)
Laurea or above	0.475	(0.292)	− 0.485	(0.552)
*Comorbidities*				
Shock	− 0.126	(0.203)	− 0.306	(0.276)
Diabetes with complications	0.394***	(0.110)	0.264*	(0.147)
Congestive heart failure	0.194***	(0.0558)	0.210***	(0.0700)
Cancer	− 0.186	(0.196)	− 0.224	(0.249)
Cerebrovascular disease	− 0.350***	(0.112)	− 0.418***	(0.144)
Pulmonary edema	− 0.0292	(0.234)	− 0.134	(0.306)
Acute renal failure	− 0.113	(0.165)	− 0.0325	(0.196)
Chronic renal failure	0.468***	(0.0650)	0.540***	(0.0806)
Cardiac dysrhythmias	− 0.286***	(0.0671)	− 0.257***	(0.0835)
*Year fixed-effects*				
2010	Reference		Reference	
2011	0.211**	(0.0853)	0.340***	(0.108)
2012	0.301***	(0.0939)	0.365***	(0.120)
2013	0.210**	(0.0970)	0.295**	(0.124)
2014	0.333***	(0.0965)	0.333***	(0.125)
2015	0.321***	(0.0921)	0.414***	(0.118)
*Regional fixed-effects*				
Piedmont (10)	Reference		Reference	
Aosta Valley (20)	− 0.547	(1.128)	− 0.116	(1.268)
Lombardy (30)	− 0.182	(0.242)	− 0.200	(0.330)
P.A. Bolzano (41)	0.0502	(0.580)	− 0.0670	(0.786)
P.A. Trento (42)	− 1.020	(0.648)	− 1.632	(0.999)
Veneto (50)	0.447**	(0.225)	0.446	(0.311)
Friuli Venezia Giulia (60)	0.121	(0.272)	0.180	(0.365)
Liguria (70)	0.173	(0.306)	0.283	(0.421)
Emilia Romagna (80)	0.899***	(0.225)	0.848***	(0.309)
Tuscany (90)	0.518***	(0.200)	0.451	(0.280)
Umbria (100)	0.141	(0.293)	0.170	(0.396)
Marche (110)	0.441*	(0.241)	0.583*	(0.310)
Lazio (120)	0.364*	(0.193)	0.0421	(0.275)
Abruzzo (130)	0.218	(0.343)	0.275	(0.457)
Molise (140)	− 1.034	(1.091)	− 0.650	(1.175)
Campania (150)	− 0.0146	(0.336)	0.00196	(0.445)
Apulia (160)	0.712**	(0.292)	0.577	(0.393)
Basilicata (170)	1.031**	(0.404)	1.225**	(0.547)
Calabria (180)	0.317	(0.372)	0.468	(0.489)
Sicily (190)	0.784*	(0.422)	0.701	(0.554)
Sardinia (200)	0.490	0.490	0.524	(0.415)

***Significant at 1%; **significant at 5%; *significant at 10%

**Table 7:** Appendix, other coefficients, Table 3

Models	All readmission		Same MDC readmission	
Variables	Coefficient	SE	Coefficient	SE
Patient characteristics (average)				
Age	− 0.00292***	(0.000752)	0.000273	(0.000312)
Male	0.0107	(0.0115)	0.00656	(0.00480)
Education	− 0.00174	(0.00305)	− 0.00201	(0.00123)
Foreign	− 0.0207	(0.0491)	− 0.0200	(0.0200)
Sum of comorbidity	− 0.0454***	(0.00636)	− 0.0117***	(0.00263)
Institution	− 0.0460	(0.0376)	− 0.0136	(0.0154)
Year (2010 reference)				
2011	0.00827	(0.00818)	0.00286	(0.00341)
2012	0.00780	(0.00861)	0.00819**	(0.00357)
2013	0.0164*	(0.00896)	0.00652*	(0.00370)
2014	0.0140	(0.00905)	0.0112***	(0.00374)
2015	0.00703	(0.00893)	0.00437	(0.00370)

***Significant at 1%; **significant at 5%; *significant at 10%
